# Numerical study of when and who will get infected by coronavirus in passenger car

**DOI:** 10.1007/s11356-022-19824-5

**Published:** 2022-03-28

**Authors:** Abd Alhamid R. Sarhan, Parisa Naser, Jamal Naser

**Affiliations:** 1 Department of Mechanical and Product Design Engineering, Swinburne University of Technology, Hawthorn, VIC 3122 Australia; 2 Johns Hopkins Bloomberg School of Public Health, Baltimore, MD 21205 USA

**Keywords:** COVID-19, Coronavirus, SARS-CoV-2, Airborne transmission, Passenger car

## Abstract

In light of the COVID-19 pandemic, it is becoming extremely necessary to assess respiratory disease transmission in passenger cars. This study numerically investigated the human respiration activities’ effects, such as breathing and speaking, on the transport characteristics of respiratory-induced contaminants in passenger car. The main objective of the present study is to accurately predict when and who will get infected by coronavirus while sharing a passenger car with a patient of COVID-19 or similar viruses. To achieve this goal, transient simulations were conducted in passenger car. We conducted a 3D computational fluid dynamics (CFD)-based investigation of indoor airflow and the associated aerosol transport in a passenger car. The Eulerian-Eulerian flow model coupled with *k*-*ε* turbulence approach was used to track respiratory contaminants with diameter ≥ 1 μm that were released by different passengers within the passenger car. The results showed that around 6.38 min, this is all that you need to get infected with COVID-19 when sharing a poorly ventilated car with a driver who got coronavirus. It also has been found that enhancing the ventilation system of the passenger car will reduce the risk of contracting Coronavirus. The predicted results could be useful for future engineering studies aimed at designing public transport and passenger cars to face the spread of droplets that may be contaminated with pathogens.

## Introduction

Although it has been nearly 2 years since the start of the coronavirus pandemic in Wuhan city, China (Lu et al. 2020; Scheuch 2020), coronavirus (COVID-19) still poses a global threat. The virus is obviously something to be scared of. It has been described as an invisible killer, a deadly pathogen, and it is difficult to control its spread. As of January 2022, there have been more than 293 M cases of COVID-19 and over 5.45 M deaths worldwide (Australia Government Department of Health 2021, April 6). The viral infection of severe acute respiratory syndrome coronavirus 2 (SARS-CoV-2) generates the coronavirus disease 2019 (COVID-19) (Coccia 2021a). The virus mainly spreads from person to person through small droplets produced by coughing, sneezing (Busco et al. 2020), and talking (Hui et al. 2019; Mittal et al. 2020; Scheuch 2020). Droplets usually fall on the ground or surface, and people can become infected by touching a contaminated surface and then touching their face. Results from earlier studies demonstrate a strong and consistent association between population density (Coccia 2020a), wind speed (Coccia 2021b), air humidity and temperature (Islam et al. 2021; Muhammad-Bashir et al. 2020), solar radiation, and other factors with the spread of the virus (Askitas et al. 2021; Wang & Huang 2021). Another critical issue related to air quality is that, according to preliminary evidence, reducing air pollution can help to control the spread of the pandemic and increase the coping capacity of infected individuals. Indeed, several studies have discovered strong links between COVID-19 transmission/mortality and high levels of air pollution. For instance, Coccia (2020b) found a strong linkage between air pollution particularly the concentration of PM10 and ozone and the vast dispersion of COVID-19 in north Italy. Another study conducted by Coccia (2020c) also found a greater number of COVID-19 cases and deaths in cities with higher air pollution levels situated in hinterland zones.

Most of the early research on this topic stated, there is no evidence on the possibility for airborne transmission SARS-CoV-2 (Leonard et al. 2020; Lu et al. 2020; Riediker & Tsai 2020; van Doremalen et al. 2020). In the middle of 2020, the possibility the virus spreading through the air was discussed by several studies (Anderson et al. 2020; Asadi et al. 2020; Li et al. 2020; Morawska & Cao 2020; Scheuch 2020; Vuorinen 2020; Yao et al. 2020). These studies provided an insightful argument about the possibility of transmission by mixing droplets containing the virus with the air and then being inhaled by a healthy individual. Transmission mechanics of infectious disease such COVID-19 in various environments are of great complexity and has been became the focus of many researchers (Abuhegazy et al. 2020; Bhattacharyya et al. 2020; Feng et al. 2020; Li et al. 2021; Vuorinen 2020). Vuorinen (2020) use high-fidelity numerical approach to investigate the aerosol transmission of SARS-CoV-2 when an infected individual cough or speak within a public indoor space. They discovered that droplets with diameters of up to 50 –100 μm could remain airborne for approximately 3 min–20 s due to rapid drying, allowing them to be inhaled by others. Lelieveld et al. (2020) developed an adjustable spreadsheet algorithm to assess the risk of COVID-19 infection from airborne transmission in indoor settings such as an office. The predicted results of this model demonstrated that there is sufficient evidence to support the hypothesis of SARS-CoV-2 aerosol transmission in indoor environments. Yan et al. (2020) proposed a numerical investigation to study the effect of the flow of cough-jet on the field of airflow and contaminants transport in a Boeing 737 cabin section. The predicted results revealed that there were up to 50% increase in the residence times and the travel distances of contaminants were also increased up to 200 μm after considering cough flow. Zhao et al. (2020) conducted a numerical study to examine the impact of the environmental conditions such as airflow velocity, temperature, and humidity on respiratory droplets generated by speech. They included in their study wide range of temperature (0 − 40 °C) and relative humidity (0 − 92%) environments. The results indicated that aerosol particles travel faster in high-humidity and low-temperature environments. The results further showed that there was an increase in the concentration of aerosol particles in low humidity and high temperature.

Traveling in passenger vehicles, such as taxis or rideshare vehicles, increases a person’s risk of contracting and spreading COVID-19 by putting people in confined spaces with others, often for extended periods of time, and exposing them to frequently touched surfaces. As a result of the urgent need to assess respiratory disease transmission in passenger cars which has been awakened by COVID-19 pandemics, this study was carefully investigated the aerosol transport of SARS-CoV-2 in a car environment using computational fluid dynamics (CFD) simulations. To the best of the authors’ knowledge, up to date, there is no CFD simulation available to predict the number of inhaled viruses within passenger car. The main goal of this study is to accurately predict the time duration to get infected while sharing a passenger car with a patient of COVID-19 or similar viruses. The present CFD model also predicts, beside the droplets’ velocity, the number of aerosol droplets inhaled by other individuals inside passenger cars. The predicted results of the present model indicate time duration to get infected and are effective in the prevention of infectious airborne diseases such as SARS-CoV-2, by identifying the movement of the droplets. These results are consistent with the evidence available in the literature which confirms transmission of COVID-19 via airborne transmission. The results of the present model could be useful for future engineering studies aimed at designing public transport and passenger cars to face the spread of droplets that may be contaminated with pathogens.

## Methodology and numerical procedures

### Mathematical model

In the present work, a 3D numerical model of airflow and the associated aerosol transport in a passenger car have been simulated with commercial CFD software AVL FIRE 2021. Eulerian method coupled with *k*-*Ɛ* model was employed in the present study to simulate the airflow field in the computational domain (i.e., passenger car). In this model, it was assumed that aerosol transport is a 2-phase flow where gas is the continuous phase and the droplets/particles are a dispersed phase. The conservation equations for the continuous (air) and dispersed (droplets) phases are discussed in following sections.

The airflow field in the computational domain is incompressible and Newtonian. The continuity equation for each phase *k* was given as follows (AVL FIRE 2021):

1 $$\frac{\partial {\mathrm{\alpha }}_{\mathrm{k}}{\uprho }_{\mathrm{k}}}{\partial \mathrm{t}}+\nabla .{\mathrm{\alpha }}_{\mathrm{k}}{\uprho }_{\mathrm{k}}{\mathbf{v}}_{\mathrm{k}}=0,\mathrm{ k}=1,\dots ,\mathrm{ N}$$

where ${\rho }_{\mathrm{k}}$ represents the density of phase $k$, ${{\varvec{v}}}_{\mathrm{k}}$ is instantaneous velocity of phase $k$ and ${\alpha }_{k}$ is the volume fraction of phase $k$. The requirement of compatibility should be observed as follows: $\sum_{\mathrm{k}=1}^{\mathrm{N}}{\mathrm{\alpha }}_{\mathrm{k}}=1$ (AVL FIRE 2021). The momentum equation for phase *k* was written as follows (AVL FIRE 2021):

2 $$\frac{\partial {\mathrm{\alpha }}_{\mathrm{k}}{\uprho }_{\mathrm{k}}{\mathbf{v}}_{\mathrm{k}}}{\partial \mathrm{t}}+\nabla .{\mathrm{\alpha }}_{\mathrm{k}}{\uprho }_{\mathrm{k}}{\mathbf{v}}_{\mathrm{k}}\otimes {\mathbf{v}}_{\mathrm{k}}=-{\mathrm{\alpha }}_{\mathrm{k}}\nabla \mathrm{p}+\nabla .{\mathrm{\alpha }}_{\mathrm{k}}\left({\uptau }_{\mathrm{k}}+{\mathbf{T}}_{\mathrm{k}}^{\mathrm{t}}\right)+{\mathrm{\alpha }}_{\mathrm{k}}{\uprho }_{\mathrm{k}}\mathbf{f}+\sum\nolimits_{\mathrm{l}=1,\mathrm{ l}\ne \mathrm{k}}^{\mathrm{N}}{\mathrm{M}}_{\mathrm{kl }}+\sum\nolimits_{\mathrm{l}=1,\mathrm{ l}\ne \mathrm{k}}^{\mathrm{N}}{\mathbf{v}}_{\mathrm{kl }}{\Gamma }_{\mathrm{kl}}\mathrm{ k}=1,\dots ,\mathrm{N}$$

here $\mathbf{f}$ represents the body force vector which comprises of gravity (**g**) and the inertial force in rotational frame $(-\upomega \times\upomega \times \mathrm{r}-2\upomega \times \mathrm{vk})$; ${\mathrm{M}}_{\mathrm{kl}}$ is the interfacial momentum interaction between phases k and l, ${\mathbf{v}}_{\mathrm{kl}}$ represents the velocity at the phase interface, and *p* is pressure (AVL FIRE 2021). The shear stress of phase k (i.e., ${\uptau }_{\mathrm{k}}$) is given as follows (AVL FIRE 2021):

3 $${\uptau }_{\mathrm{k}}= {\upmu }_{\mathrm{i}}^{\mathrm{t}}\left[\left(\nabla {\otimes \mathbf{v}}_{\mathrm{k}}+{\left(\nabla {\otimes \mathbf{v}}_{\mathrm{k}}\right)}^{\mathrm{T}}\right)-\frac{2}{3}\nabla .{\mathbf{v}}_{\mathrm{k}}\mathbf{I}\right]$$

here ${\upmu }_{\mathrm{k}}$ represents the molecular viscosity. Reynolds stress, ${\mathbf{T}}_{\mathrm{k}}^{\mathrm{t}}$, is computed as follows (AVL FIRE 2021):

4 $${\mathbf{T}}_{\mathrm{k}}^{\mathrm{t}}=-{\uprho }_{\mathrm{k}}\overline{{\mathbf{v} }_{\mathrm{k}}^{\mathrm{^{\prime}}}{\mathbf{v}}_{\mathrm{k}}^{\mathrm{^{\prime}}}}={\upmu }_{\mathrm{i}}^{\mathrm{t}}\left[\left(\nabla {\otimes \mathbf{v}}_{\mathrm{k}}+{\left(\nabla {\otimes \mathbf{v}}_{\mathrm{k}}\right)}^{\mathrm{T}}\right)-\frac{2}{3}\nabla .{\mathbf{v}}_{\mathrm{k}}\mathbf{I}\right]-\frac{2}{3}{\uprho }_{\mathrm{i}}{\mathrm{k}}_{\mathrm{i}}\mathbf{I}$$

In the present model the turbulent viscosity, ${\upmu }_{\mathrm{i}}^{\mathrm{t}}$, was computed as follows (AVL FIRE 2021):

5 $${\upmu }_{\mathrm{k}}^{\mathrm{t}}={\mathrm{C}}_{\upmu }{\uprho }_{\mathrm{k}}\frac{{{\mathrm{K}}_{\mathrm{k}}}^{2}}{{\upvarepsilon }_{\mathrm{k}}}$$

where ${\mathrm{K}}_{\mathrm{k}}$ represents kinetic energy and ${\upvarepsilon }_{\mathrm{k}}$ is dissipation rate of energy. In this work, the following equations were employed to calculate the turbulence dissipation rate and turbulence kinetic energy (AVL FIRE 2021):

6 $$\frac{\partial {\mathrm{\alpha }}_{\mathrm{k}}{\uprho }_{\mathrm{k}}{\mathbf{v}}_{\mathrm{k}}}{\partial \mathrm{t}}+\nabla .{\mathrm{\alpha }}_{\mathrm{k}}{\uprho }_{\mathrm{k}}{\mathbf{v}}_{\mathrm{k}}{\mathrm{k}}_{\mathrm{k}}= \nabla .{\mathrm{\alpha }}_{\mathrm{k}}\left({\upmu }_{\mathrm{k}}+\frac{{\upmu }_{\mathrm{k}}^{\mathrm{t}}}{{\upsigma }_{\mathrm{k}}}\right)\nabla {\mathrm{k}}_{\mathrm{k}}+{\mathrm{\alpha }}_{\mathrm{k}}{\mathrm{P}}_{\mathrm{k}}-{\mathrm{\alpha }}_{\mathrm{k}}{\uprho }_{\mathrm{k}}{\upvarepsilon }_{\mathrm{k}}+\sum\nolimits_{\mathrm{l}=1,\mathrm{j}\ne \mathrm{k}}^{\mathrm{N}}{\mathrm{K}}_{\mathrm{kl}}+\sum\nolimits_{\mathrm{l}=1,\mathrm{l}\ne \mathrm{k}}^{\mathrm{N}}{{\mathrm{k}}_{\mathrm{kl}}\Gamma }_{\mathrm{kl}}\mathrm{ k}=1,\dots ,\mathrm{ N}$$

7 $$\frac{\partial {\mathrm{\alpha }}_{\mathrm{k}}{\uprho }_{\mathrm{k}}{\mathbf{v}}_{\mathrm{k}}}{\partial \mathrm{t}}+\nabla .{\mathrm{\alpha }}_{\mathrm{k}}{\uprho }_{\mathrm{k}}{\mathbf{v}}_{\mathrm{k}}{\mathrm{k}}_{\mathrm{k}}= \nabla .{\mathrm{\alpha }}_{\mathrm{k}}\left({\upmu }_{\mathrm{k}}+\frac{{\upmu }_{\mathrm{k}}^{\mathrm{t}}}{{\upsigma }_{\upvarepsilon }}\right)\nabla {\upvarepsilon }_{\mathrm{k}}+\sum\nolimits_{\mathrm{l}=1,\mathrm{j}\ne \mathrm{k}}^{\mathrm{N}}{\mathrm{D}}_{\mathrm{kl}}+\sum\nolimits_{\mathrm{l}=1,\mathrm{l}\ne \mathrm{k}}^{\mathrm{N}}{\upvarepsilon }_{\mathrm{kl}}{\Gamma }_{\mathrm{kl}}+{\mathrm{\alpha }}_{\mathrm{k}}{\mathrm{C}}_{1}{\mathrm{P}}_{\mathrm{k}}\frac{{\upvarepsilon }_{\mathrm{k}}}{{\mathrm{k}}_{\mathrm{k}}}-{\mathrm{\alpha }}_{\mathrm{k}}{\mathrm{C}}_{2}{\uprho }_{\mathrm{k}}\frac{{{\upvarepsilon }_{\mathrm{k}}}^{2}}{{\mathrm{k}}_{\mathrm{k}}}+{\mathrm{\alpha }}_{\mathrm{k}}{\mathrm{C}}_{4}{\uprho }_{\mathrm{k}}{\upvarepsilon }_{\mathrm{k}}\nabla .{\mathbf{v}}_{\mathrm{k}}\mathrm{ k}=1,\dots ,\mathrm{ N}$$

The standard values of all empirical constants in the *k*-*ɛ* turbulence model are *C*_1_ = 1.44, *C*_2_ = 1.92, *C*_4_ = -0.373, σ_k_ = 1.0, σ_ε_ = 1.3, *C*_μ_ = 0.09 and σ_T_ = 0.9 (AVL FIRE 2021).

Turbulent dispersion force and drag force have a significant role in the momentum interfacial exchange between phases. In this model, the momentum interfacial exchange between continuous phase (air/gas) and dispersed phase (droplets/particles) are taken into account. The momentum interfacial exchange is written as follows (AVL FIRE 2021):

8 $${\mathbf{M}}_{\mathrm{c}}={\mathrm{C}}_{\mathrm{D}}\frac{1}{8}{\uprho }_{\mathrm{c}}{\mathrm{A}}_{\mathrm{i}}^{\mathrm{^{\prime}}\mathrm{^{\prime}}\mathrm{^{\prime}}}\left|{\mathbf{v}}_{\mathrm{r}}\right|{\mathbf{v}}_{\mathrm{r}}+{C}_{TD}{\rho }_{c}{k}_{c}\nabla {\alpha }_{d}=-{\mathbf{M}}_{\mathrm{d}}$$

The subscripts d and c are respectively the dispersed and continuous phases. The relative velocity is expressed as: ${\mathbf{v}}_{\mathrm{r}}={\mathbf{v}}_{\mathrm{d}}-{\mathbf{v}}_{\mathrm{c}}$. The interfacial area for the flow is calculated as follows (AVL FIRE 2021):

9 $${\mathrm{A}}_{\mathrm{i}}^{\mathrm{^{\prime}}\mathrm{^{\prime}}\mathrm{^{\prime}}}={\left(36\uppi \right)}^\frac{1}{3}{\mathrm{N}}^{\mathrm{^{\prime}}\mathrm{^{\prime}}\mathrm{^{\prime}}\frac{1}{3}}{\mathrm{\alpha }}_{\mathrm{d}}^\frac{2}{3}$$

where the number density, N′′′, was calculated from the cavitation mass exchange model. The drag coefficient ${\mathrm{C}}_{\mathrm{D}}$ was calculated as a function of droplets terminal velocity and presented as follows (system Cs 2020):

10 $${C}_{D}=\frac{4}{3}\mathrm{g }{\mathrm{d}}_{\mathrm{p}}\frac{\left[{\rho }_{p}-{\rho }_{\mathrm{g}}\right]}{{\rho }_{\mathrm{g}} {\mathrm{Vo}}^{2}}$$

where ${\rho }_{p}$ and ${\rho }_{\mathrm{g}}$ are respectively density of droplets and gas phase, d_p_ represents the diameter of the droplet, and v_o_ is the terminal velocity of droplets. Gravity, which causes particle sedimentation, is regarded as an important physical mechanism for removing droplets from room air. This mechanism depends on the droplets size and its terminal velocity. The size of the droplet used in the present investigation was 1 µm and their terminal velocity was calculated at room temperature (i.e., 20 °C) (Scheuch 2020, system Cs 2020).

### Model description and computational setup

**Fig. 1:**
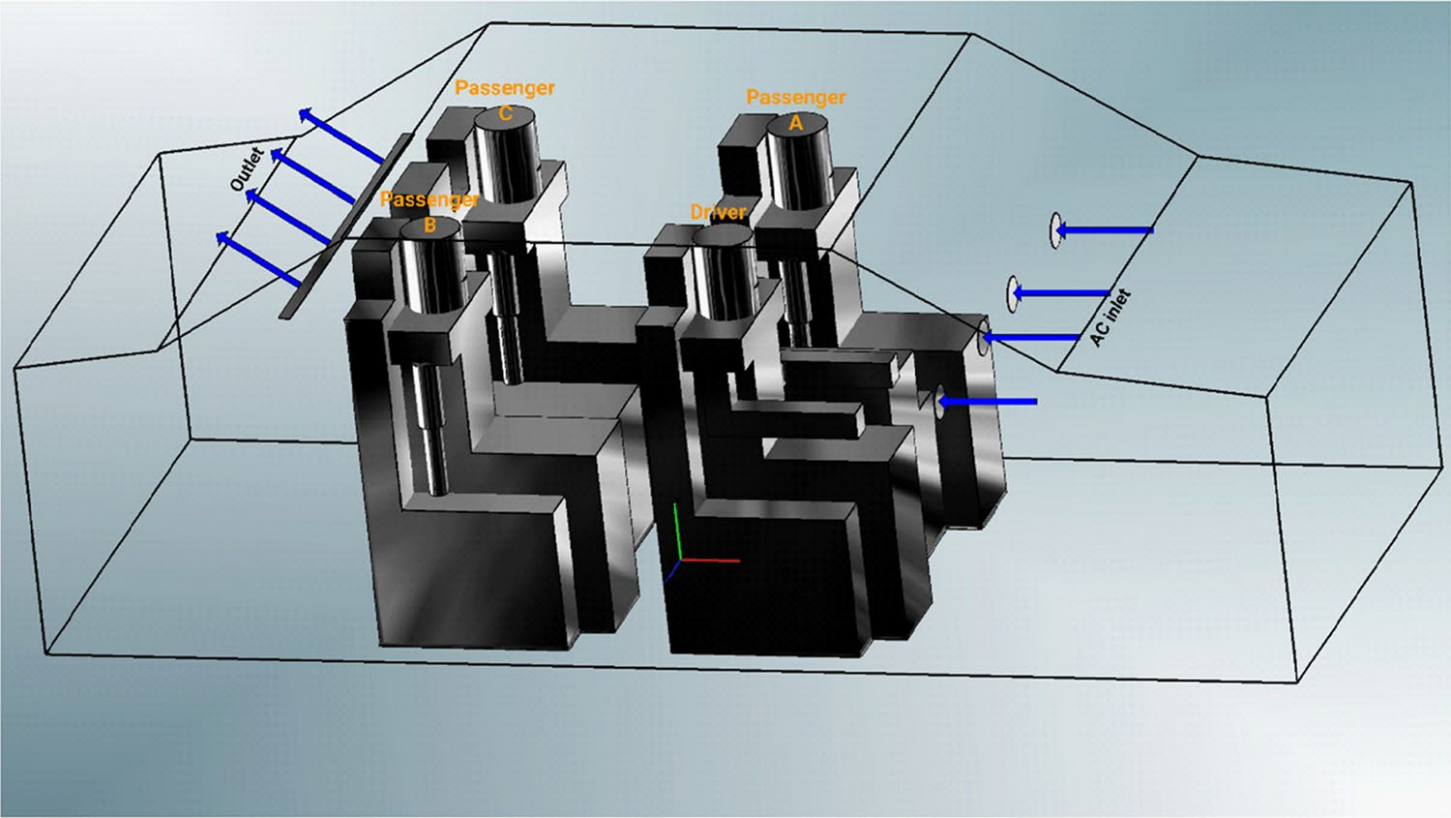
Computational model of car cabin section and passengers

Human respiration activities, such as breathing and speaking within passenger car, has been simulated in this model. The present study utilized CFD software AVL FIRE 2021. The computational domain was assumed to be a medium-sized passenger car which illustrated in Fig. 1. In addition to the driver, three passengers were occupying the car, we labelled them as follows: Driver, Passenger A, Passenger B, and Passenger C (please see Fig. 1).

Since the purpose of the current investigation is to identify the safest spot within the passenger car while sharing it with a patient of COVID-19, the infected person’s location is referred in the paper as index case. For each case, four different modes of HVAC system were used. The present model examined four different scenarios which are listed in Table 1.

**Table 1:** List of case student in the present work

	Index case	Modes of HAVC system				
		Fan speed	Level 1	Level 2	Level 3	Level 4
Case 1	Driver	air speed (m/s)	1.38	2.6	4	5.88
Case 2	Passenger A	air speed (m/s)	1.38	2.6	4	5.88
Case 3	Passenger B	air speed (m/s)	1.38	2.6	4	5.88
Case 4	Passenger C	air speed (m/s)	1.38	2.6	4	5.88

**Fig. 2:**
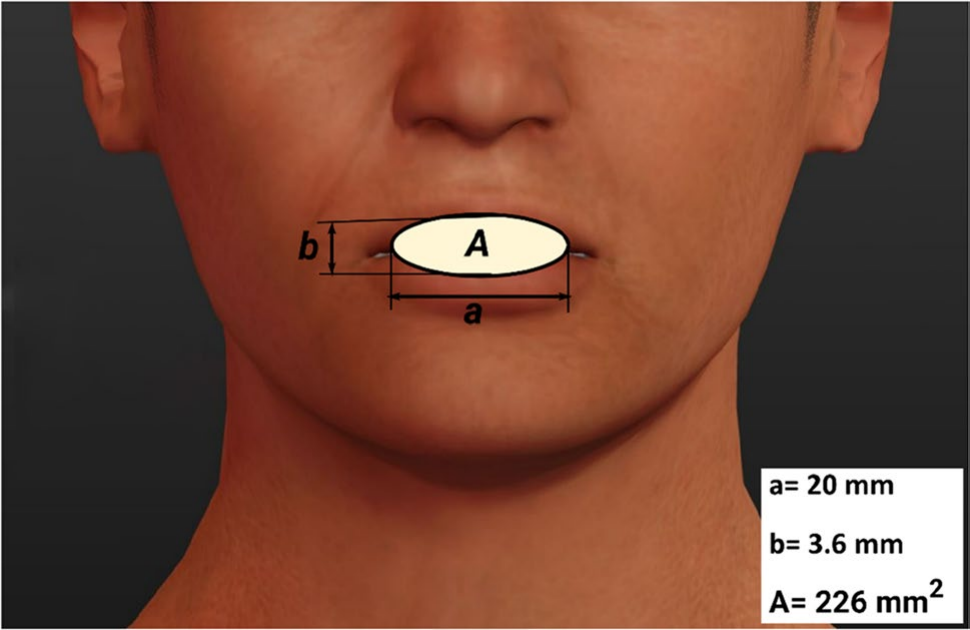
Mouth opening shape and dimensions

In the present model, human respiration activities, such as breathing and speaking within the car cabin has been simulated with CFD. With regard to breathing mode, the infected and non-infected individuals are modelled to be breathing 10 times per minute with a pulmonary rate of 6 L/min with a sinusoidal cycle (3-s inhalation + 3-s exhalation) (Hui et al. 2019; Riediker & Tsai 2020, Vuorinen 2020). In the present model, exhalation and inhalation has been assumed to be through the mouth only. Fig. 2 shows the geometrical aspects of the mouth opening of manikins used in this model.

The present investigation considered air and droplets/particles as separate phases. To date, there is very little information available in literature about the number of coronaviruses produced by COVID-19-infected subject. However, evidence showed that the size of SARS-CoV-2 (i.e., 60 –160 nm) is very close to the size of influenza viruses (80–100 nm) (Centers for Disease Control and Prevention (CDC) (2021), March 21, Mittal et al. 2020; Morawska & Cao 2020; Nikitin et al. 2014; Scheuch 2020; World Health Organization (WHO) (2021), 25 May). Researchers stated that the concentration number of aerosol droplets in human exhaled breath $\sim$ 10,000 droplets per liter (Fabian et al. 2008; Nikitin et al. 2014). Therefore, one exhaled breath, which is between 0.3 and 0.75 l (Ai & Melikov 2018) could contain an order of 10^3^ droplets (≤ 1 µm) (Yan et al. 2018). These droplets are small enough to remain suspended in the air and pose a risk of airborne transmission. The average size of the droplets was assumed to be 1.0 µm (Anderson et al. 2020; Asadi et al. 2019; Fairchild and Stampfer 1987; Yip et al. 2019).

To account for the domain’s transient dynamic situation, the transport equations for all phases throughout the computational domain are solved. Various grid independency tests with different mesh resolutions were carried out in order to achieve acceptable accuracy while maintaining an acceptable computational time. No significant impact of the grid resolutions on the predicted results was found.

### Numerical procedures

The commercial software AVL FIRE 2021 based on the finite volume method (FVM) method was used in the present model to solve the main equations (i.e., momentum, continuity, turbulence, and scalar) of the multi-phase flow within the computational domain. The first order upwind scheme was used to discretise to discretise the governing equations. In comparison to the second-order scheme, which is computationally expensive, the accuracy of the first scheme is reasonable (Sarhan et al. 2017). To obtain accurate results of turbulent flows, turbulence models were employed. The solution was obtained using a pressure-based solver, and the pressure was determined using the SIMPLE algorithm (Patankar & Spalding 1983). For all simulation cases, the process was solved using a transient simulation with a total duration of 3600 s and time steps of 0.01 s. Table 2 provides a summary of the numerical procedures.

**Table 2:** Summary of the model formulation

General	Linear solver type	GSTB	
	Pressure formulation	SIMPLE	
	Run mode	Unsteady,$\Delta t=0.01\mathrm{s}$	
	End time	3600 s	
	Gravitational body force	Full body force-Y direction	
	Convergence criteria	0.0001	
	Inlet condition	Normal velocity	
	Outlet condition	Static pressure, 100,000 Pa	
	Mesh type	950,595 grids, symmetric grid	
Models	Eulerian approarch		
	Drag model	Schiller-Neuman	
	Viscous-standard-$k-\varepsilon$,dispresed		
Control	Number of phases = 2	air/gas and droplets	
	Continuous phase = gas phase		
	Secondary phase = droplets	${\mathrm{d}}_{\mathrm{p}}\le$ 1 $\mathrm{\mu m}$	
	Minimum volume fraction	1E − 006	
Materials	Gas = air		
	droplets = water		
Solver control	Discretization	Calculation of boundary values	Extrapolate
		Calculation of derivative	Least sq. fit
	Equation control	Compressibility	Incompressible
		Wall treatment	Hybrid wall treatment
	Differencing scheme	Momentum	First-order UDS
		Continuity	First-order UDS
		Turbulence	First-order UDS
		Energy	First-order UDS
		Scalar	First-order UDS
		Volume fraction	First-order UDS
No. of iterations 5			

### Initial and boundary conditions

In the computational domain (i.e., car cabin), the volume fraction of the gas phase is set to one and the volume fraction of the droplets is set to zero. The boundary conditions have a significant impact on the accuracy of the flow computation and how well it represents the physical situation. The boundary conditions used in this model are inlet, outlet, and wall. The normal velocity of air flow at the mouth surface of each individual was described by sinusoidal function which fit quite well with normal human breathing process (Leonard et al. 2020). While for the HVAC system, constant normal velocity was used as an inlet boundary condition. At the outlet, atmospheric pressure has been used. For both phases (i.e., air & droplets), no-slip wall boundary conditions are set at the walls. (i.e., air & droplets). Figure 1 shows boundary conditions used in the present study.

## Results and discussions

**Fig. 3:**
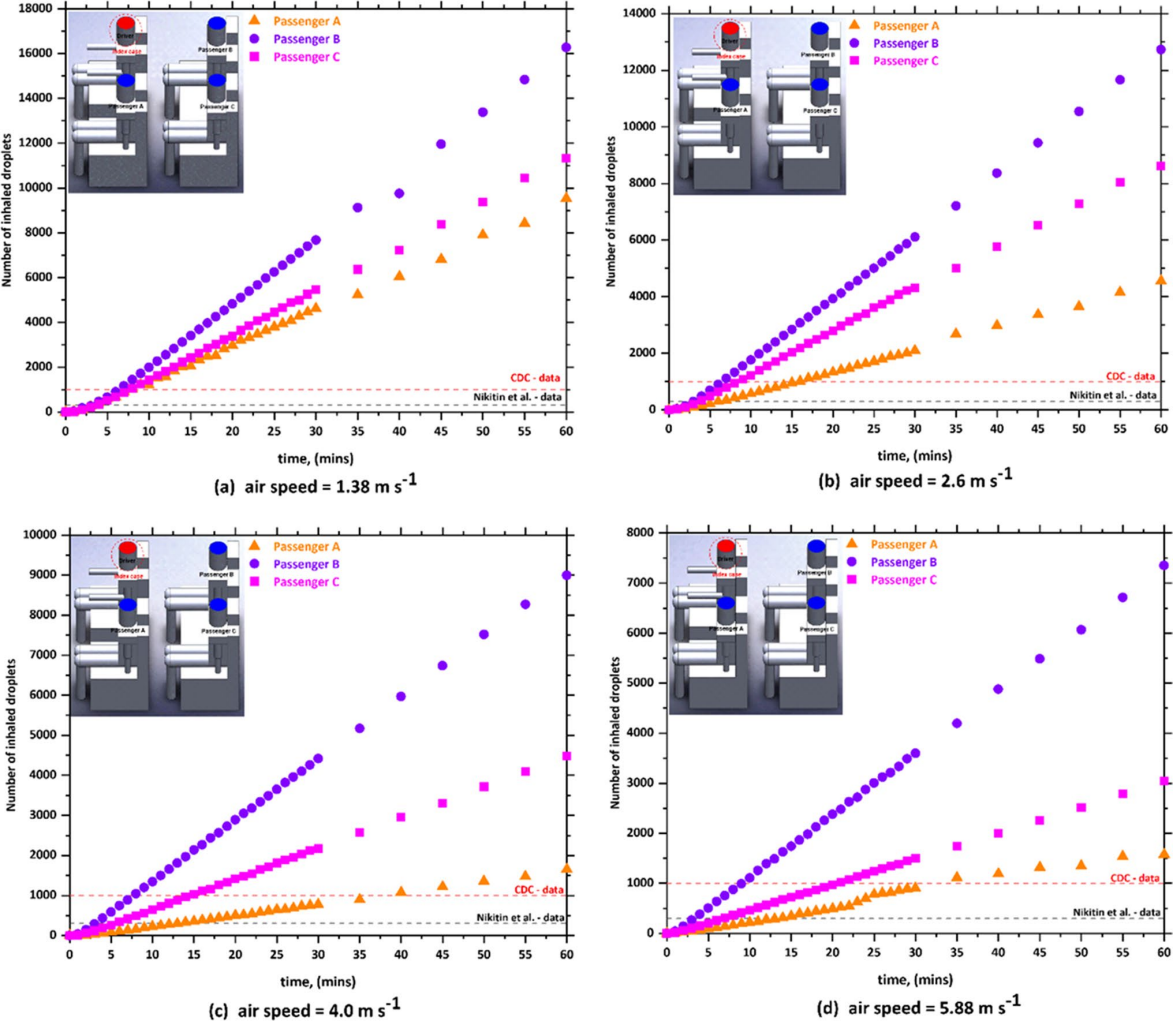
The number of inhaled droplets/particles by healthy individuals in a car cabin–case 1 (driver is the index case)

**Fig. 4:**
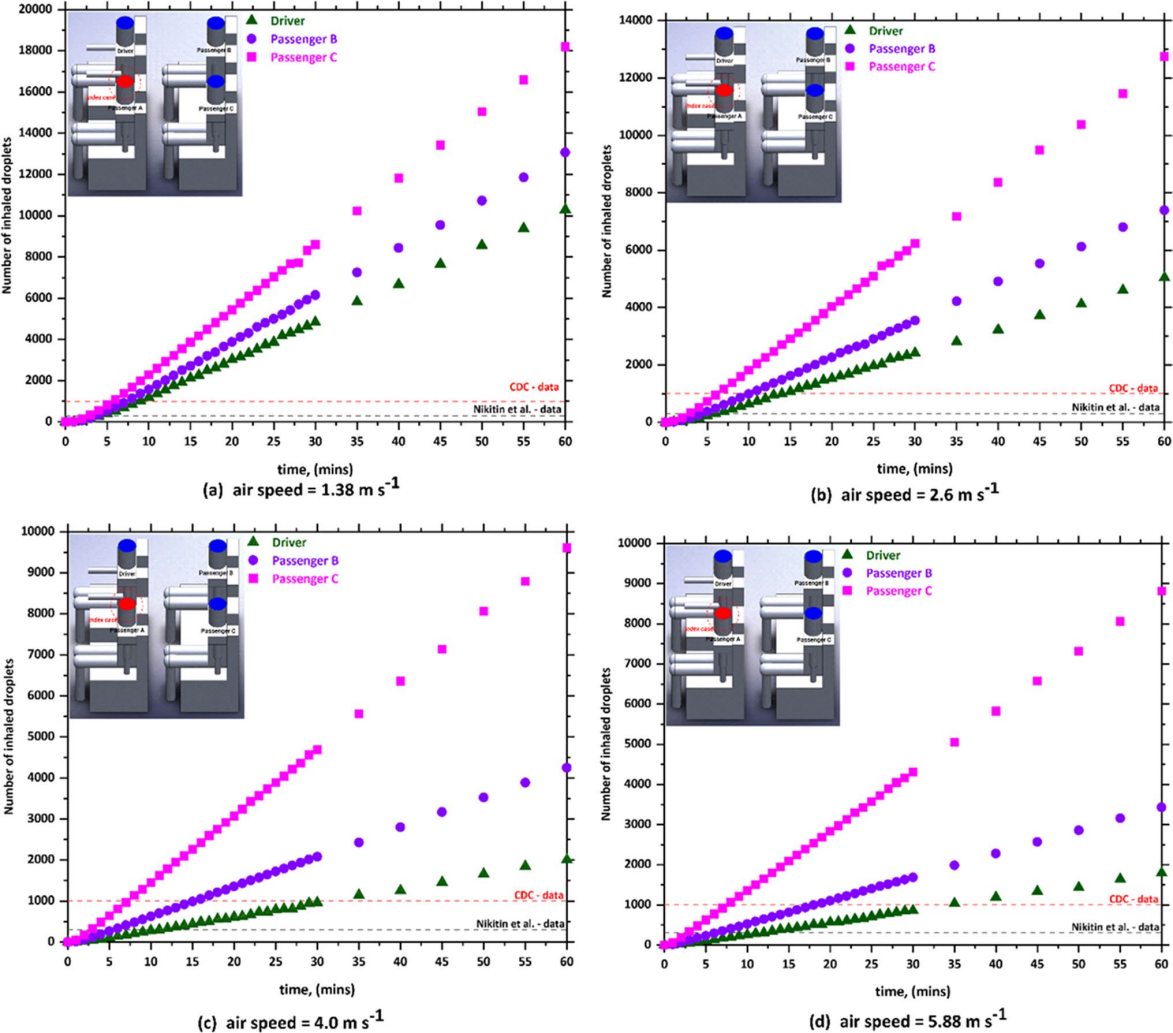
The number of inhaled droplets/particles by healthy individuals in a car cabin–case 2 (passenger A is the index case)

**Fig. 5:**
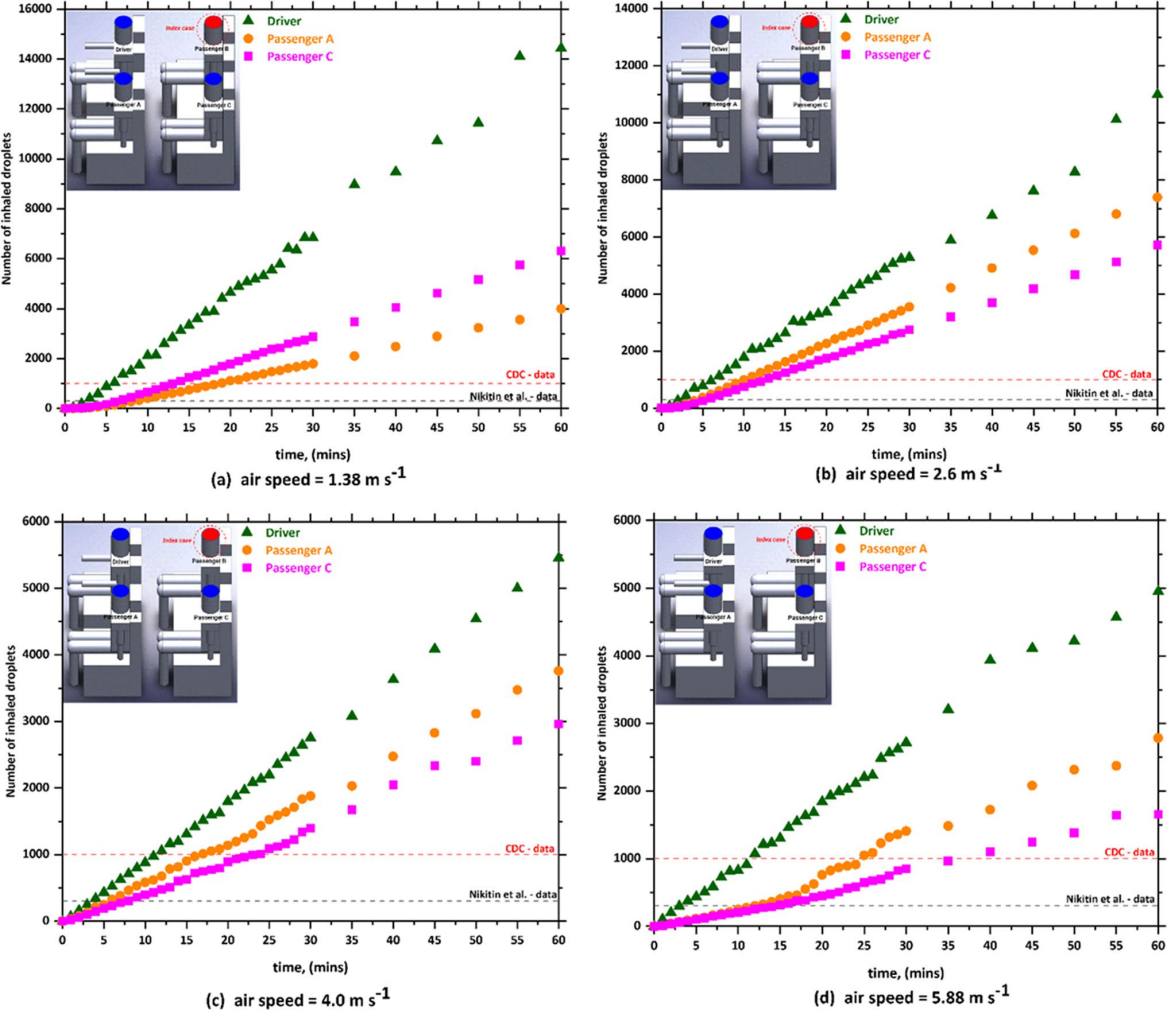
The number of inhaled droplets/particles by healthy individuals in a car cabin–case 3 (passenger B is the index case)

**Fig. 6:**
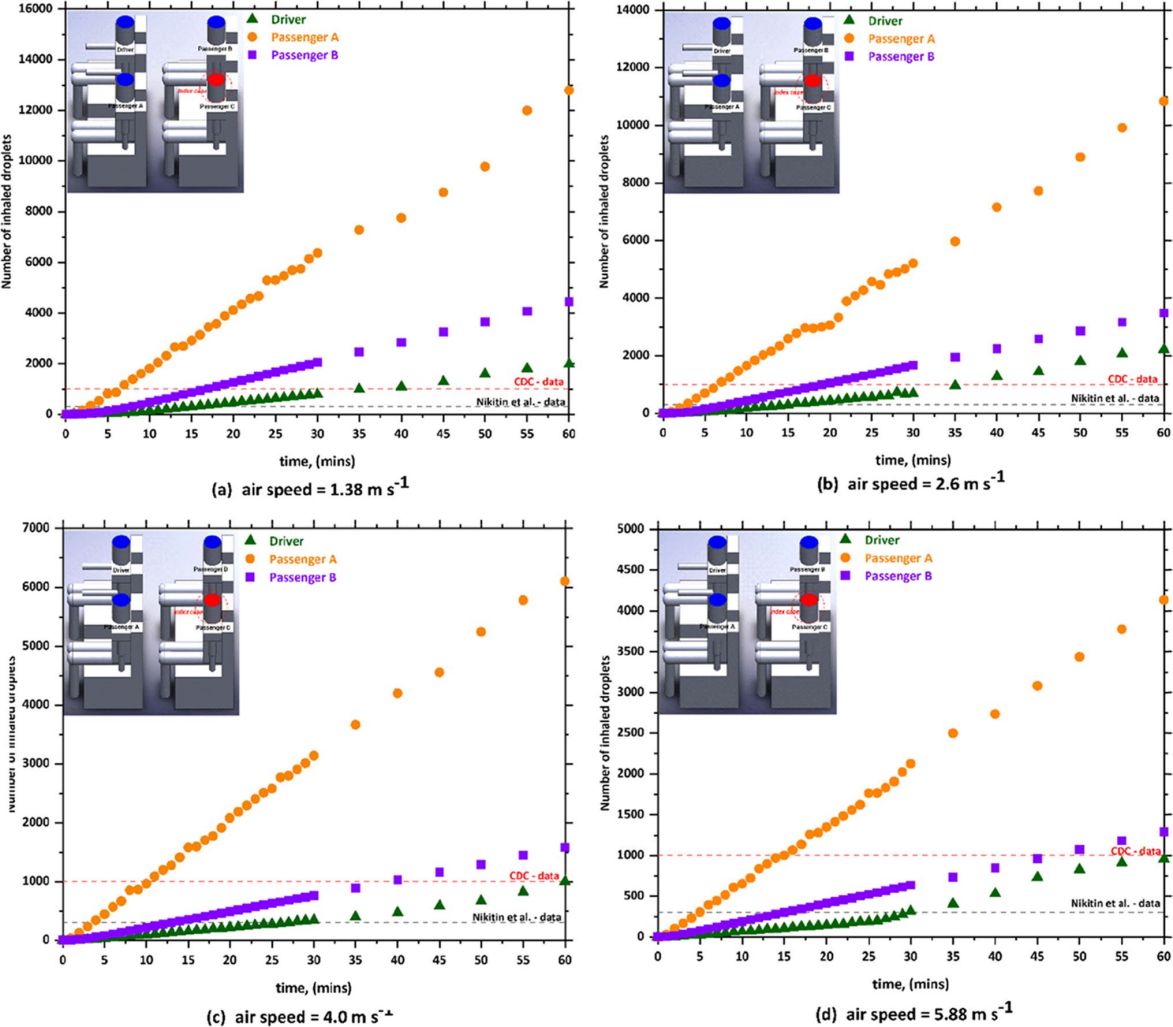
The number of inhaled droplets/particles by healthy individuals in a car cabin–case 4 (passenger C is the index case)

Although there is no available data on how many SARS-CoV-2 particles come out with every breath of an infected individual, the ability to predict the number of inhaled aerosol droplets, which comes out of infected individuals, will give us an indication on the probability of contracting COVID-19. It is not necessarily that all aerosols droplets would carry viruses, however, part of them could. The present CFD model predicts the number of aerosol droplets, exhaled by infected individual, and inhaled by other individuals inside the car cabin through breathing and speaking. The predicted results of the simulations for the passenger car are plotted in Figs. 3, 4, 5 and 6. As already mentioned, they consist in scenario 1 (driver is the index case), scenario 2 (passenger a is the index case), scenario 3 (passenger B is the index case), and scenario 4 (passenger C is the index case). In each one of these scenarios, different modes of HVAC system were employed (i.e., (a) *v*_1_ = 1.38, (b) *v*_2_ = 2.6, (c*)* v_3_ = 4, and (c) *v*_4_ = 5.88 m s^−1^.).

The x–y plots represent the number of contaminated aerosols droplets inhaled by different healthy subjects as a consequence of the breathing and speaking events. Such comparison allows investigating the airborne contagion exposure for all the individuals sitting in the car cabin. Figure 3 shows the predicted results obtained from case 1 (i.e., drivers is the index case). It can be seen from the data in Fig. 3a-d that the most likely passenger to inhale more contaminated aerosols droplets is passenger B who is sitting directly behind the driver (i.e., index case). While passenger A who is sitting beside the driver (i.e., index case) will inhale fewer droplets compared with the other passengers within the vehicle. For example, at *v*_1_ = 1.38 m s^−1^, passenger B inhaled more than 16,270 aerosol droplets after 60 min. While, for the same mode of HVAC setting and for the same period of time, passenger A inhaled about 9530 droplets. Looking at the data obtained from case 2 (i.e., passenger A is the index case), again we can see the passenger C who is sitting directly behind the index case (i.e., passenger A) will inhale the highest number of contaminated droplets. While the driver will inhale a lower number of contaminated droplets compared with other passengers, for the same mode of the HVAC system, passenger C inhaled around 18,200 droplets, whereas the driver inhaled about 10,290 droplets within 60 min. This result may be explained by the fact that the airflow from the HVAC system will carry most of the contaminated droplets to the back seats that will cause the passenger who is sitting directly behind the index case to inhale the highest number of contaminated droplets.

The predicted results obtained from case 3 (i.e., passenger B is the index case) is shown in Fig. 5a-d. In case 3, the driver was sitting directly in front of the index case. It can be seen from this figure that the driver will inhale the highest number of contaminated droplets compared with other passengers. The same trend was observed in case 5 (i.e., passenger C is the index case), where a passenger who is sitting in front of the index case inhaled (i.e., passenger A is the index case) the highest number of contaminated droplets. This result could be attributed to the fact that the exhalation of the infected person loaded with contaminated droplets will move directly to the front seats. This will cause that the person who is sitting directly in front of the infected person will inhale more contaminated droplets.

By comparing the results obtained from the different cases, we found that the worst scenario was when the infected person is sitting directly behind the driver where this will cause that the driver will inhale about 48,000 contaminated droplets. The exhalation of the infected person loaded with contaminated droplets will move directly to the front where it collides with the air current coming from the cooling system which leads to circulating the contaminated air current for a longer period of time around the driver and this ultimately will lead to the increase of contaminated droplets inhaled by the driver. The other observation we can make from Figs. 3, 4, 5, and 6 is that the number of inhaled contaminated droplets increases with the increase of the duration of the trip. This finding is expected since the infected person will exhale more contaminated droplets into the car cabin.

**Fig. 7:**
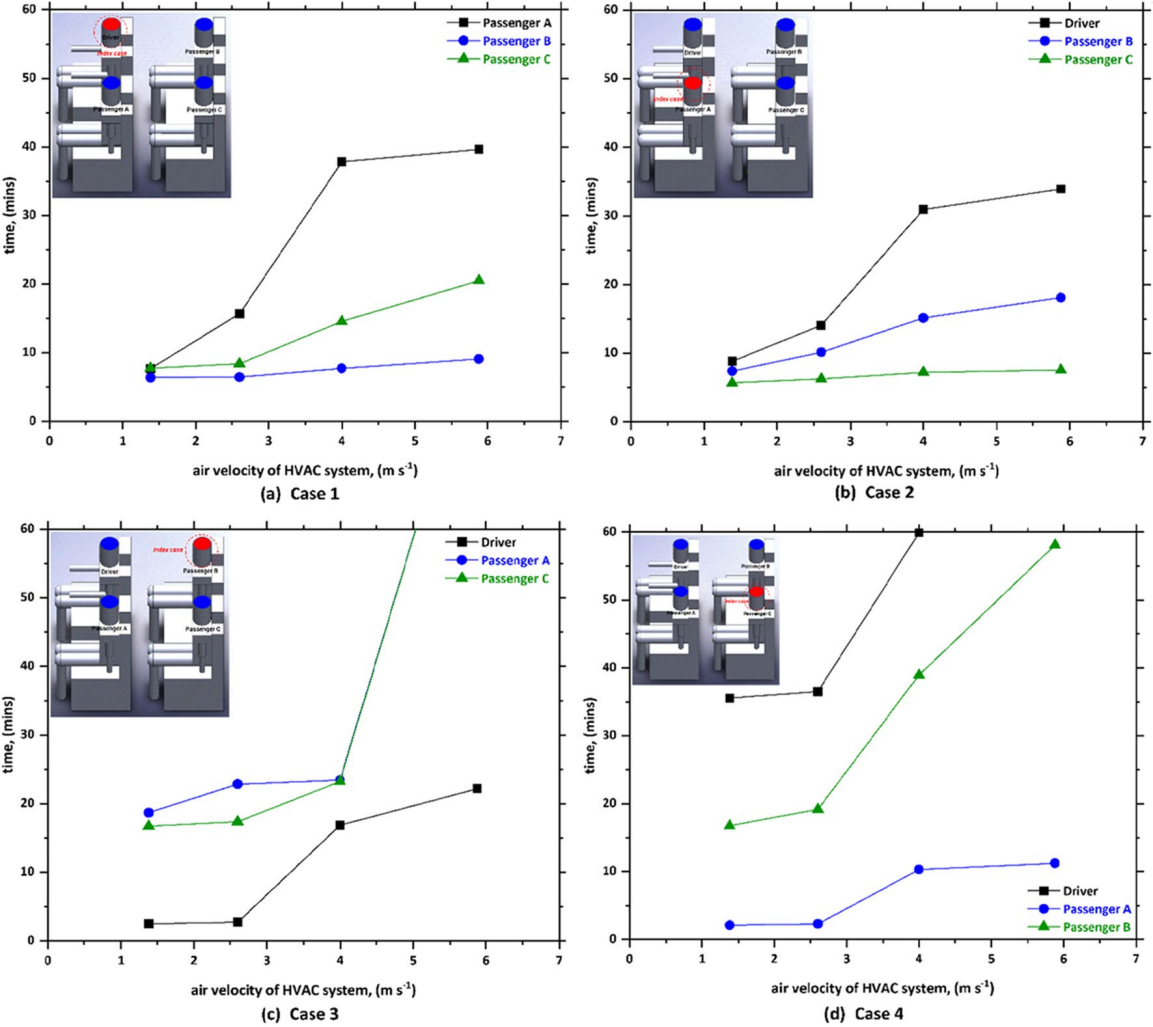
The effect of air velocity of HVAC system on the time required to inhale 1000 droplets

The effect of air velocity of the HVAC system on the time required to inhale 1000 droplets by other healthy passengers within a car cabin for case 1, case 2, case 3, and case 4 is given in Fig. 7. The obtained results suggest that the HVAC system mode has a marked effect on the number of inhaled contaminated droplets. Figure 7 graph shows that there has been a notable increase in the time required to inhale 1000 droplets with the increase of air velocity of the HVAC system, for all cases studied in the present work. For case 1 (i.e., driver is the index case), the time required passenger A to inhale 1000 droplets increases from 7.76 s at *v*_1_ = 1.38 m s^−1^ to approximately 39.65 s at *v*_4_ = 1.38 m s^−1^. Similar trend was observed for the other cases. We can also see from Fig. 7 that the effect of the mode HVAC system will become more significant when the case is sitting in the back seats of the vehicle (please refer to Fig. 7c and d). For example, in the third case (i.e., passenger B is the index case), even after 60 min, the driver and passenger will not inhale 1000 droplets which means there is a high chance these two (i.e., driver and passenger C) will not get infected with the COVID-19. Figure 7d also shows a similar trend where the increase in the air velocity of the HVAC system will lead to a considerable increase in the time required to inhale 1000 droplets. We can see from Fig. 7d that the time increased from 16.76 s at *v*_1_ = 1.38 m s^−1^ to 58.07 s at *v*_4_ = 5.88 m s^−1^. From the data in Fig. 7d, it can also be seen that the driver will inhale much less than 1000 droplets even after 60 min, which again means there is a high possibility that the driver will not contract the virus.

**Fig. 8:**
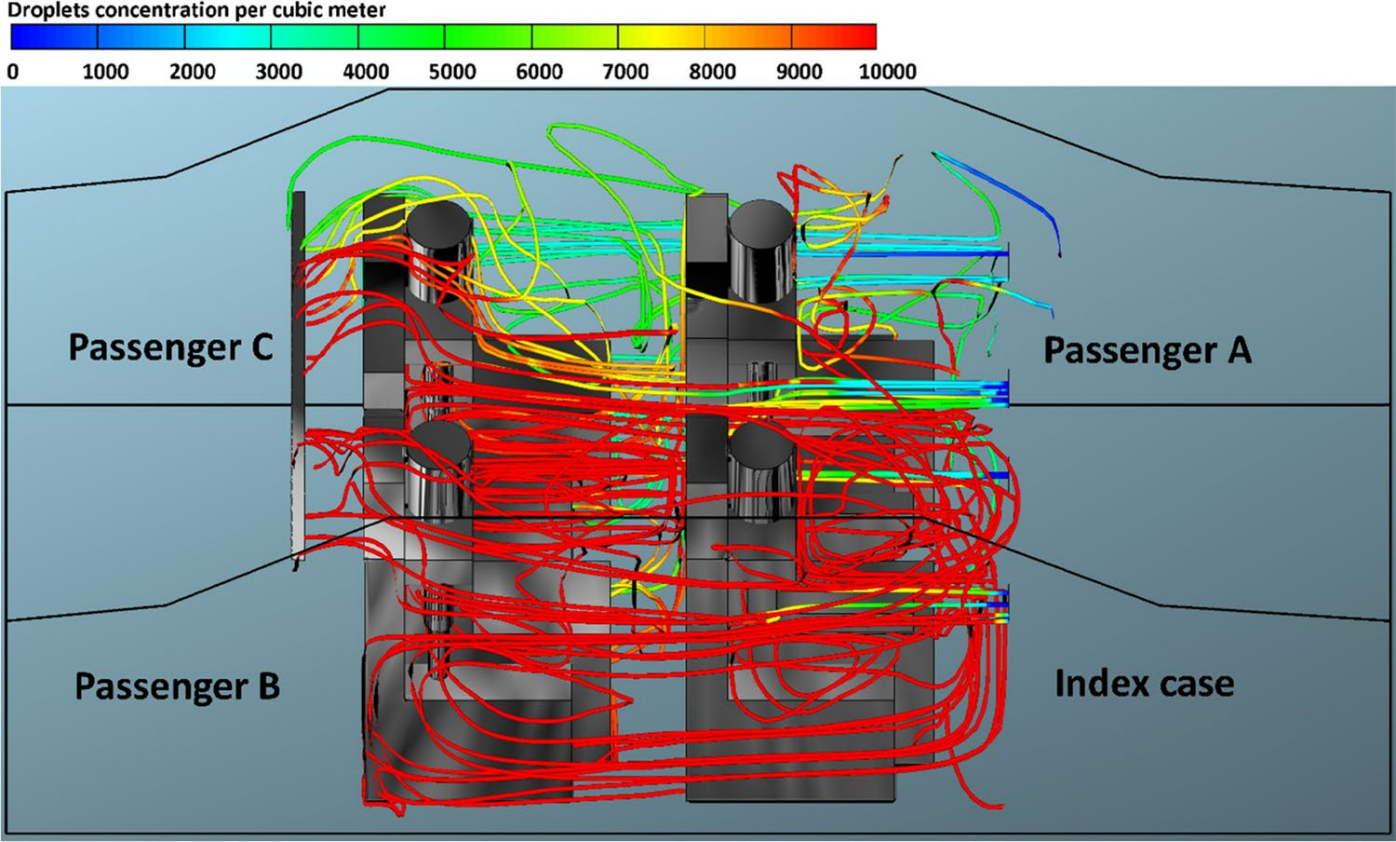
Concentration of contaminated droplets per cubic meter available in the car cabin for case 1 and *v*_1_ = 1.38 m s^−1^

**Fig. 9:**
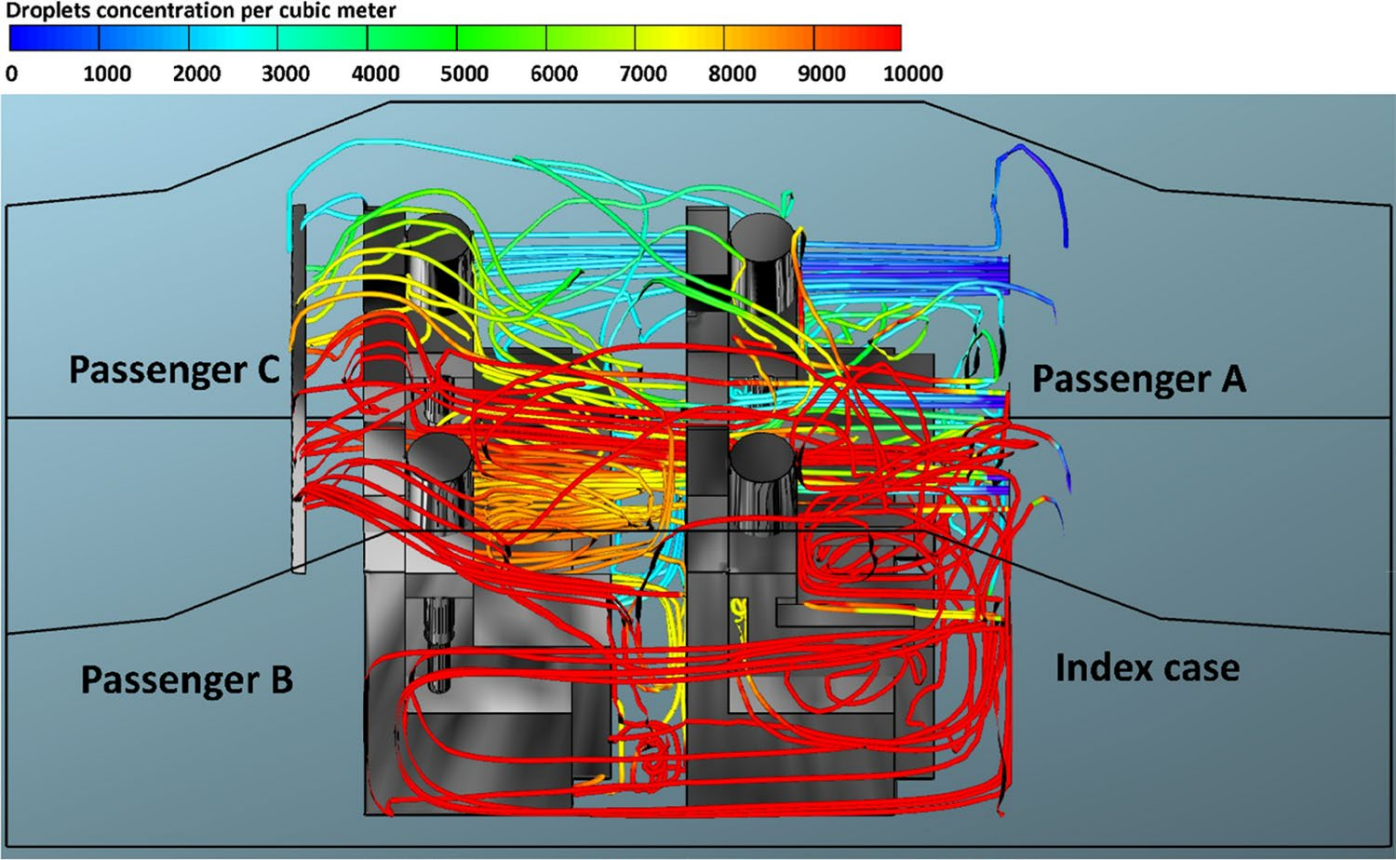
Concentration of contaminated droplets per cubic meter available in the car cabin for case 1 and *v*_2_ = 2.6 m s^−1^

**Fig. 10:**
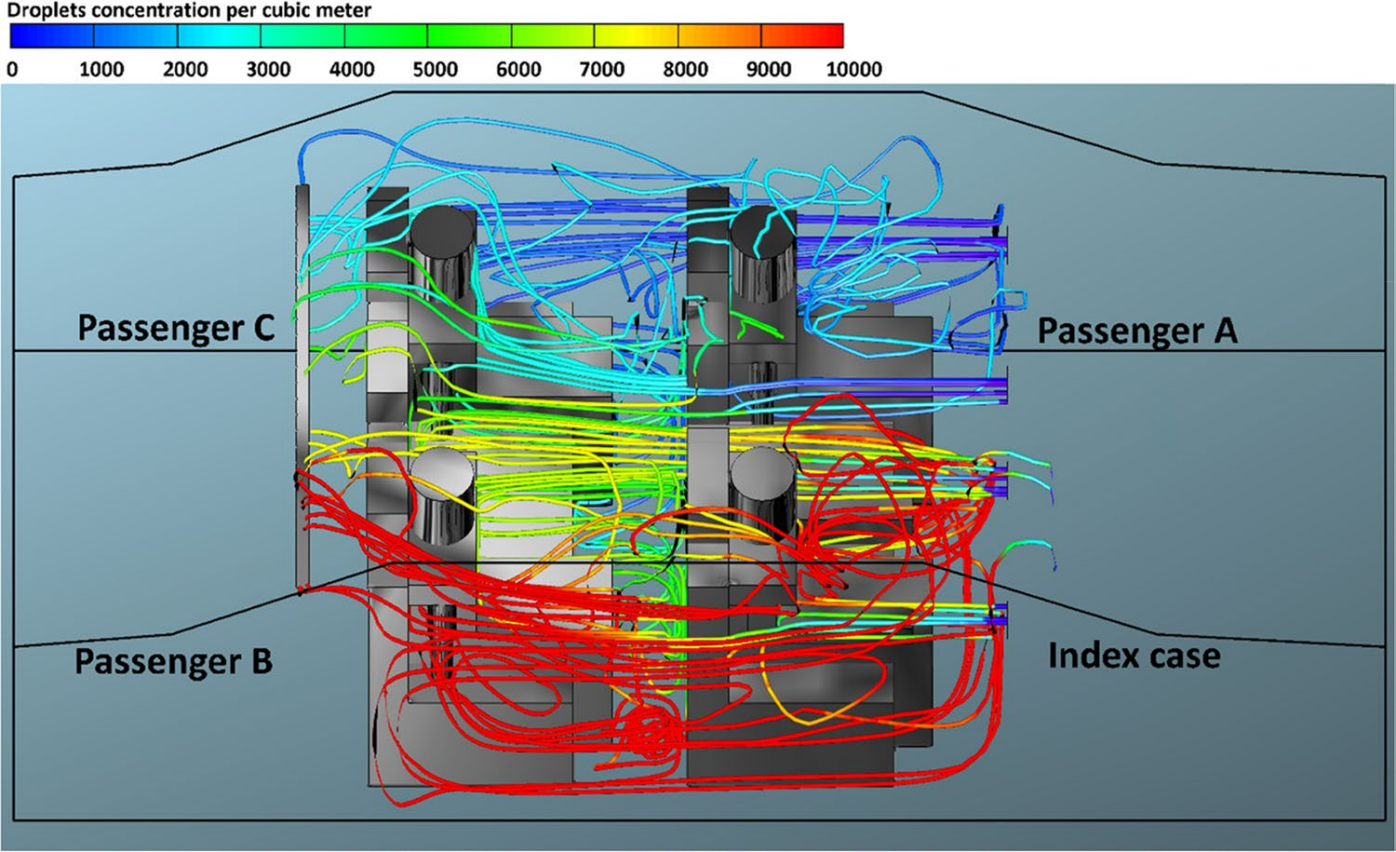
Concentration of contaminated droplets per cubic meter available in the car cabin for case 1 and *v*_3_ = 4 m s^−1^

**Fig. 11:**
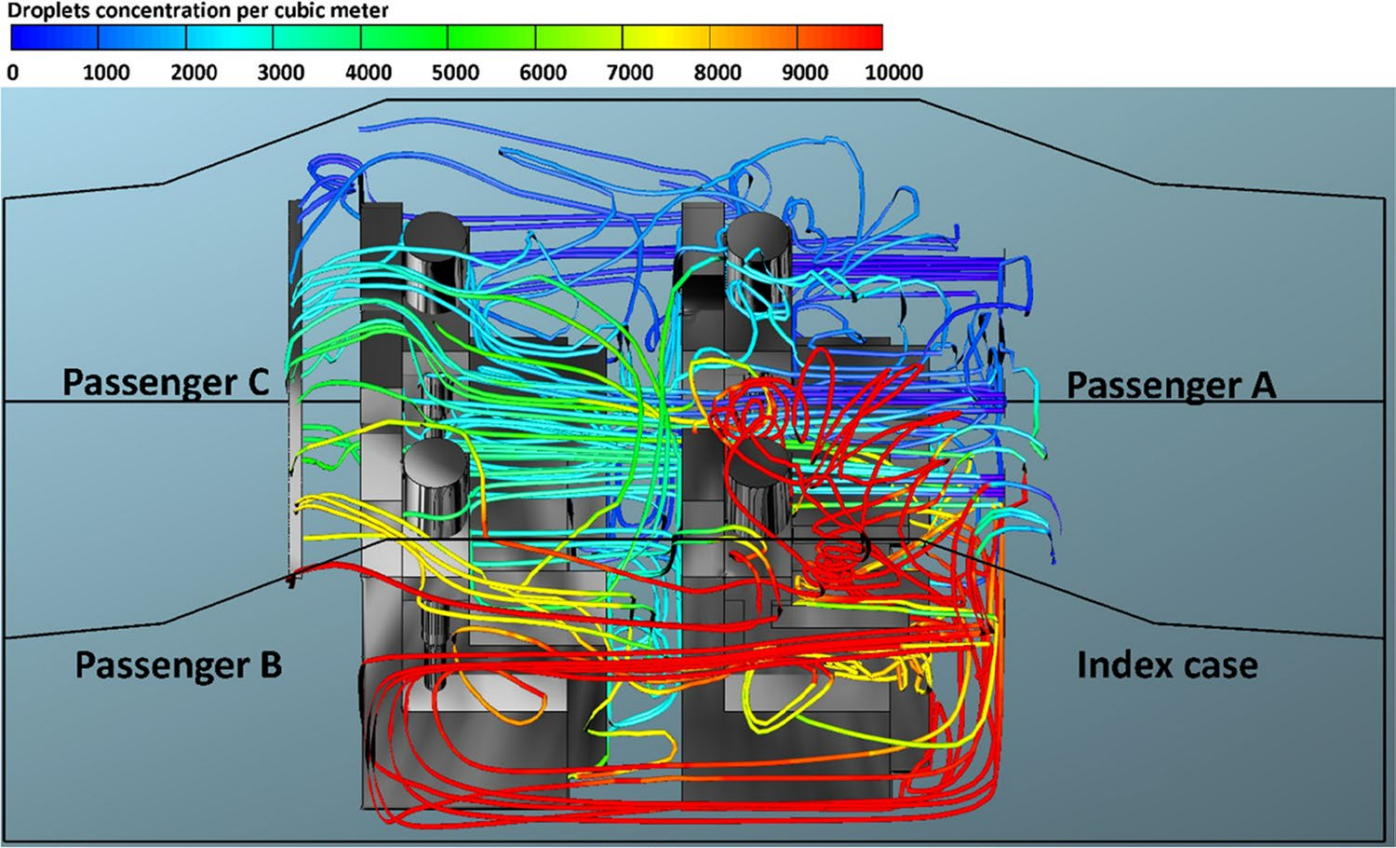
Concentration of contaminated droplets per cubic meter available in the car cabin for case 1 and *v*_4_ = 5.88 m s^−1^

**Fig. 12:**
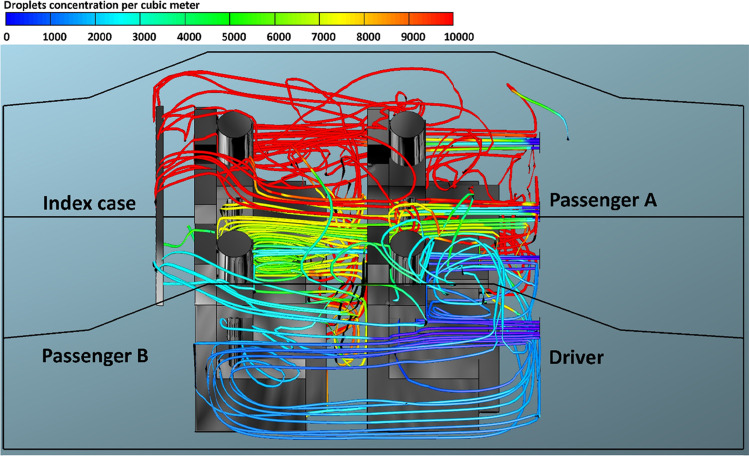
Concentration of contaminated droplets per cubic meter available in the car cabin for case 4 and *v*_1_ = 1.38 m s^−1^

**Fig. 13:**
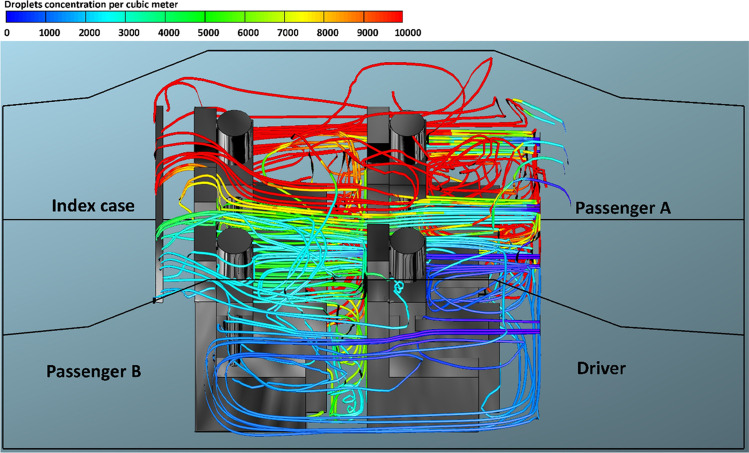
Concentration of contaminated droplets per cubic meter available in the car cabin for case 4 and *v*_2_ = 2.6 m s^−1^

**Fig. 14:**
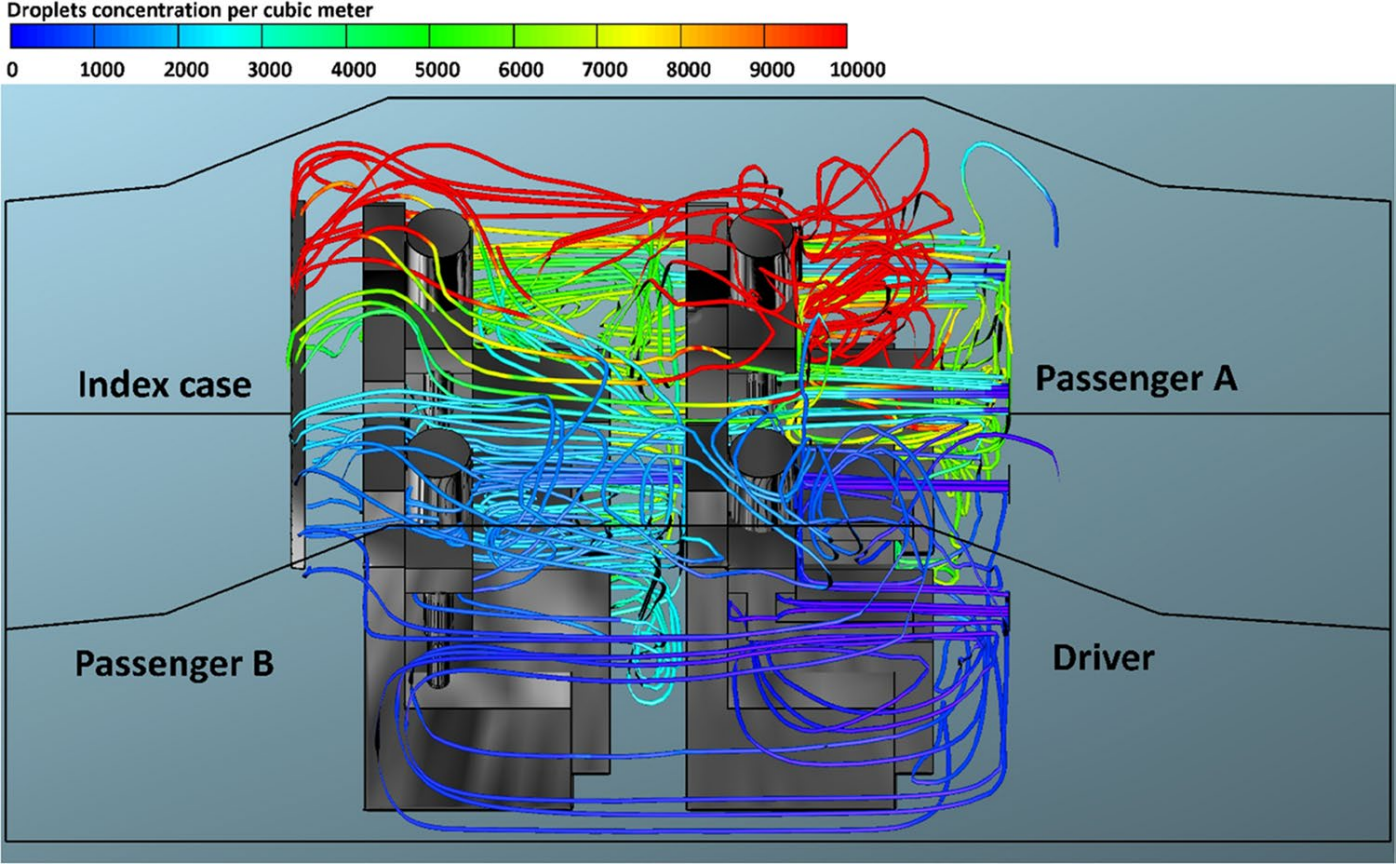
Concentration of contaminated droplets per cubic meter available in the car cabin for case 4 and *v*_3_ = 4 m s^−1^

**Fig. 15:**
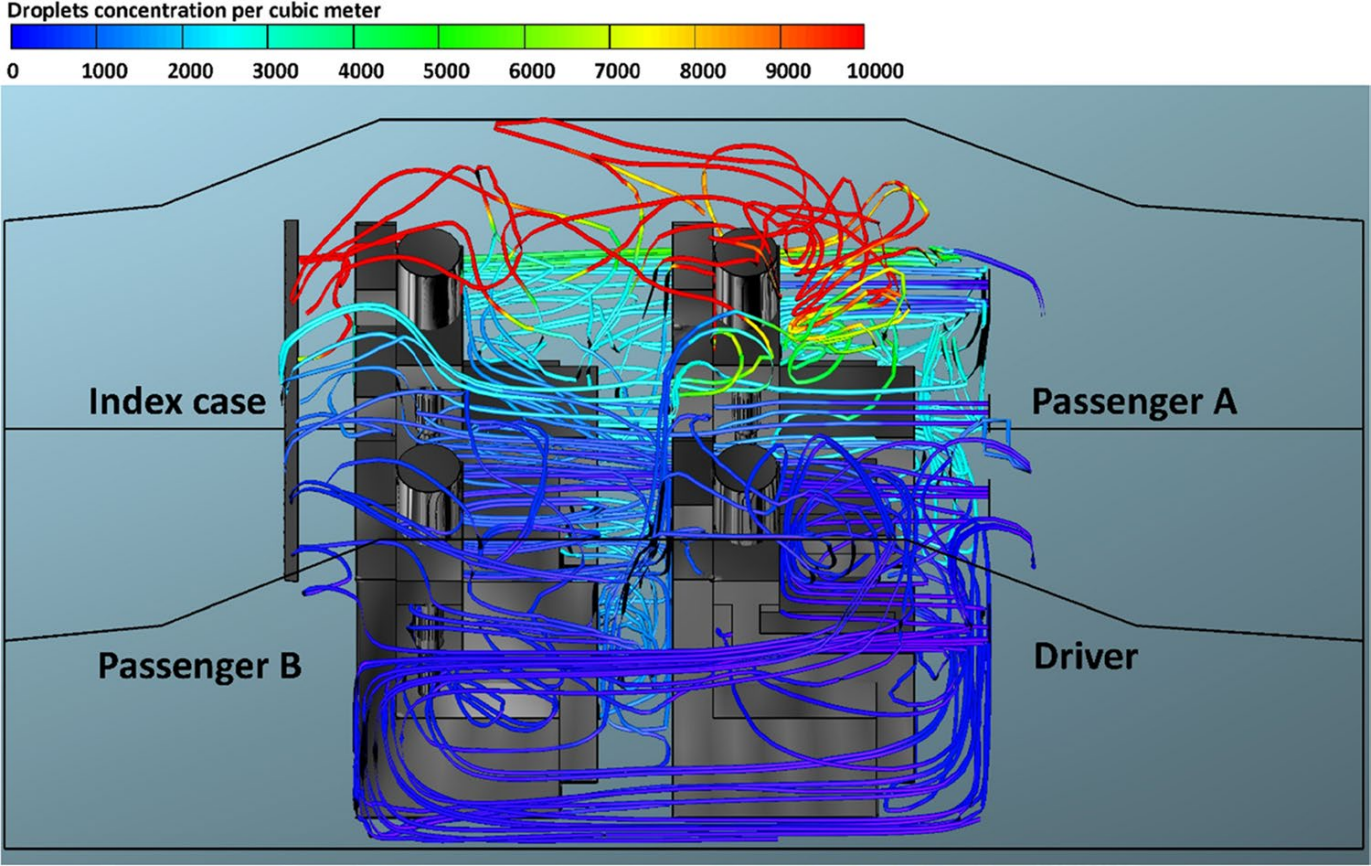
Concentration of contaminated droplets per cubic meter available in the car cabin for case 4 and *v*_4_ = 5.88 m s^−1^

The present model treated air and droplets as two different independent phases. The use of this assumption enables us to track the volume fraction of the droplet phase throughout the computational domain and thus predict the number of contaminated droplets in the car cabin. The predicted droplet concentrations per cubic meter exhaled from the index case for case 1 and case 4, and for different mode of the HVAC system are presented in Figs. 8–15. We can see from these figures the contaminated cloud envelope in the computational domain (i.e., car cabin), where the color scale at the top of the figures illustrates the concentration number of contaminated droplets per cubic meter.

Figure 8 shows the predicted results of droplet concentrations per cubic meter exhaled from the index case (driver) for case 1 and *v*_1_ = 1.38 m s^−1^. Figure 8 reveals that the stream of exhaled droplets from the index case (driver) collides with the air stream of the of the HVAC system. The high momentum of the HVAC system stream pushes the contaminated air current toward the back seats. This air movement led to the circulation of aerosol droplets inside the computational, and this explains the high number of contaminated droplets that passenger B, who is sitting directly behind the driver, inhaled (please, refer to Fig. 3). The red color refers to a higher concentration of contaminated droplets in that part of the computational domain which leads to an increase in the possibility of inhaling more contaminated droplets and thus increases the risk of contracting the infection. The predicted results of droplet concentrations per cubic meter exhaled from the index case (driver) for case 1 and different modes of the HVAC system (air velocity = 2.6, 4.0, and 5.88 m s^−1^) are shown in Figs. 9, 10, and 11. Here also we can see that the concentration of the contaminated droplets per cubic meter in the right half of the car cabin at the driver side is much higher than the second half of the vehicle (i.e., the left half of the car cabin). It can be seen from the data in Figs. 9, 10, and 11 that the air stream of the HVAC system has similar influence on the concentration of contaminated droplets. However, the concentration of contaminated droplets decreases with increase of air velocity of the HVAC system. The observed decrease in the concentration of contaminated droplets could be attributed to the increase in the amount of fresh air coming through the HVAC unit from outside the car cabin. This fresh air will partially replace the contaminated air by pushing it outside the car through the ventilation system. The amount of fresh air will increase with the increase in the air velocity of the HVAC unit thus will cause more reduction in the concentration of the contaminated droplets within the car cabin. This effect explains the reduction in the number of inhaled droplets by the healthy passengers with the increase in the air velocity of the HVAC unit (please refer to Fig. 3).

Figures 12, 13, 14, and 15 illustrate the effect of air velocity of the HVAC system on the number of contaminated droplets per cubic meter exhaled from the index case (passenger C) though normal breathing and talking in 3600 s (case 4). Different levels of air velocities were employed: *v*_1_ = 1.38, *v*_2_ = 2.6, *v*_3_ = 4.0 & *v*_4_ = 5.88 m s^−1^, respectively. Figure 12 shows that the concentration of the contaminated droplets per cubic meter near passengers A and C (the left section of the car cabin) is highest in comparison with second half of the car cabin (the right section of the car cabin). These results are likely to be related to the influence of the air currents coming from the HVAC system. The air current coming from the HVAC system collided with the stream of exhaled droplets from the index case (passenger C). The high momentum of the HVAC system streams pushes the contaminated air currents again toward the back seats near the index case (passenger C). More contaminated droplets will suspend into the air stream and travel back to the front seat. This circulation movement of the air stream will result in an increase in the concentration of the contaminated droplets with the car cabin especially near passengers A and C. These results explain the high number of inhaled droplets by passenger A in case 4 (please see Fig. 6). Figures 13, 14, and 15 show the concentration of contaminated droplets for case 4 and at *v*_2_ = 2.6, *v*_3_ = 4.0 & *v*_4_ = 5.88 m s^−1^. Again, it can be seen that the increase in air velocity t of the HVAC system will lead to a decrease in concentration of contaminated droplets. As explained in the previous section, this reduction can be attributed to the increase in the amount of fresh coming through the HVAC unit from outside the car.

## Summary and conclusions

In this work, Computational fluid dynamics (CFD) was utilized to study aerosol transport of SARS-CoV-2 in a car cabin. The developed CFD model was utilized to accurately predict the time duration to get infected while sharing passenger car with a patient of COVID-19 or similar viruses. The present CFD model predicts the number of aerosol droplets inhaled by every individual inside the car cabin through breathing and speaking. Predictions of this type are useful for the effective prevention of infectious airborne diseases like SARS-CoV-2, by identifying the movement of the droplets in confined spaces like passenger car and predict the spread of disease. The following are the main findings of the current study:

- 6.38 min, this is all that you need to get infected with COVID-19 when sharing a poorly ventilated car with a driver who got Coronavirus.
- If you were infected with coronavirus, sitting in the rear passenger seat will reduce the risks of transmitting the infection to the driver.
- The safest place for you to use a passenger car with an unknown driver, like a taxi driver, is the passenger seat beside the driver.
- Enhancing the ventilation system of the passenger car will reduce the risk of contracting coronavirus.

It is worthy to mention that the present model ignored the droplets evaporation. This is a deficiency of the present model, as droplet evaporation plays a singularly important role in the eventual fate of a droplet. The droplet evaporation rate increases with higher temperature and lower relative humidities, which is not the case in the present model. A further study with more focus on droplets evaporation is therefore suggested.

## Data Availability

The data that support the findings of this study are available on request from the authors.

## References

[CR1] Abuhegazy M, Talaat K, Anderoglu O, Poroseva SV (2020). Numerical investigation of aerosol transport in a classroom with relevance to COVID-19. Phys Fluids (1994).

[CR2] Ai ZT, Melikov AK (2018). Airborne spread of expiratory droplet nuclei between the occupants of indoor environments: a review. Indoor Air.

[CR3] Anderson EL, Turnham P, Griffin JR, Clarke CC (2020). Consideration of the aerosol transmission for COVID-19 and public health. Risk Anal.

[CR4] Asadi S, Wexler AS, Cappa CD, Barreda S, Bouvier NM, Ristenpart WD (2019). Aerosol emission and superemission during human speech increase with voice loudness. Sci Rep.

[CR5] Asadi S, Bouvier N, Wexler AS, Ristenpart WD (2020): The coronavirus pandemic and aerosols: does COVID-19 transmit via expiratory particles? Aerosol Sci. Technol. 0, 1–4 10.1080/02786826.2020.1749229 PMC7157964 32308568

[CR6] Askitas N, Tatsiramos K, Verheyden B (2021). Estimating worldwide effects of non-pharmaceutical interventions on COVID-19 incidence and population mobility patterns using a multiple-event study. Sci Rep.

[CR7] Australia Government Department of Health (2021): What you need to know about coronavirus (COVID-19). Department of Health

[CR8] AVL FIRE (2021): Software Documentation, Graz, Austria

[CR9] Bhattacharyya S, Dey K, Paul AR, Biswas R (2020). A novel CFD analysis to minimize the spread of COVID-19 virus in hospital isolation room. Chaos Solitons Fractals.

[CR10] Busco G, Yang SR, Seo J, Hassan YA (2020). Sneezing and asymptomatic virus transmission. Phys Fluids (1994).

[CR11] Centers for Disease Control and Prevention (CDC) (2021, March 21): About COVID-19

[CR12] Coccia M (2020). An index to quantify environmental risk of exposure to future epidemics of the COVID-19 and similar viral agents: theory and practice. Environ Res.

[CR13] Coccia M (2020). Factors determining the diffusion of COVID-19 and suggested strategy to prevent future accelerated viral infectivity similar to COVID. Sci Total Environ.

[CR14] Coccia M (2020). How (un) sustainable environments are related to the diffusion of COVID-19: the relation between coronavirus disease 2019, air pollution, wind resource and energy. Sustainability.

[CR15] Coccia M (2021). The impact of first and second wave of the COVID-19 pandemic in society: comparative analysis to support control measures to cope with negative effects of future infectious diseases. Environ Res.

[CR16] Coccia M (2021). How do low wind speeds and high levels of air pollution support the spread of COVID-19?. Atmos Pollut Res.

[CR17] Fabian P, McDevitt JJ, DeHaan WH, Fung RO, Cowling BJ, Chan KH, Leung GM, Milton DK (2008). influenza virus in human exhaled breath: an observational study. PLoS One.

[CR18] Fairchild C, Stampfer J (1987). Particle concentration in exhaled breath. Am Ind Hyg Assoc J.

[CR19] Feng Y, Marchal T, Sperry T, Yi H (2020). Influence of wind and relative humidity on the social distancing effectiveness to prevent COVID-19 airborne transmission: A numerical study. J Aerosol Sci.

[CR20] Hui DS, Chow BK, Lo T, Tsang OTY, Ko FW, Ng SS, Gin T, Chan MTV (2019). exhaled air dispersion during high-flow nasal cannula therapy versus CPAP via different masks. Eur Respir J.

[CR21] Islam T, Pitafi AH, Arya V, Wang Y, Akhtar N, Mubarik S, Xiaobei L (2021). Panic buying in the COVID-19 pandemic: a multi-country examination. J Retail Consum Serv.

[CR22] Lelieveld J, Helleis F, Borrmann S, Cheng Y, Drewnick F, Haug G, Klimach T, Sciare J, Su H, Poschl U (2020). model calculations of aerosol transmission and infection risk of COVID-19 in indoor environments. Int J Environ Res Public Health.

[CR23] Leonard S, Strasser W, Whittle JS, Volakis LI, DeBellis RJ, Prichard R, Atwood CW, Dungan GC (2020). Reducing aerosol dispersion by high flow therapy in COVID-19: High resolution computational fluid dynamics simulations of particle behavior during high velocity nasal insufflation with a simple surgical mask. J Am Coll Emerg Physicians Open.

[CR24] Li Y, Qian H, Hang J, Chen X, Cheng P, Ling H, Wang S, Liang P, Li J, Xiao S, Wei J, Liu L, Cowling BJ, Kang M (2021). Probable airborne transmission of SARS-CoV-2 in a poorly ventilated restaurant. Build Environ.

[CR25] Li Y, Qian H, Hang J, Chen X, Hong L, Liang P, Li J, Xiao S, Wei J, Liu L, Kang M (2020): Evidence for probable aerosol transmission of SARS-CoV-2 in a poorly ventilated restaurant. medRxiv, 2020.04.16.20067728

[CR26] Lu J, Gu J, Li K, Xu C, Su W, Lai Z, Zhou D, Yu C, Xu B, Yang Z (2020). COVID-19 outbreak associated with air conditioning in restaurant, Guangzhou, China, 2020. Emerg Infect Dis.

[CR27] Mittal R, Ni R, Seo J-H (2020). The flow physics of COVID-19. J Fluid Mech.

[CR28] Morawska L, Cao J (2020). Airborne transmission of SARS-CoV-2: The world should face the reality. Environ Int.

[CR29] Muhammad-Bashir S, Al-Oufi M, Al-Hakami M, Nadeem M, Mudiyanselage K, Idriss H (2020). Comparison between the performance of high concentrated and non-concentrated PV-cells for hydrogen production using PEM water electrolyzers. Sol Energy.

[CR30] Nikitin N, Petrova E, Trifonova E, Karpova O (2014): Influenza virus aerosols in the air and their infectiousness. Advances in virology 2014 10.1155/2014/859090 PMC4147198 25197278

[CR31] Patankar SV, Spalding DB (1983): PAPER 5 - a calculation procedure for heat, mass and momentum transfer in three-dimensional parabolic flows. In: Patankar SV, Pollard A, Singhal AK , Vanka SP (Editors), Numerical Prediction of Flow, Heat Transfer, Turbulence and Combustion. Pergamon, pp. 54–73

[CR32] Riediker M, Tsai DH (2020). Estimation of viral aerosol emissions from simulated individuals with asymptomatic to moderate coronavirus disease 2019. JAMA Netw Open.

[CR33] Sarhan AR, Naser J, Brooks G (2017). Bubbly flow with particle attachment and detachment – a multi-phase CFD study. Sep Sci Technol.

[CR34] Scheuch G (2020). Breathing is enough: for the spread of influenza Virus And SARS-CoV-2 by breathing only. J Aerosol Med Pulm Drug Deliv.

[CR35] system Cs (2020): Particle Size & Settling Velocities

[CR36] van Doremalen N, Bushmaker T, Morris DH, Holbrook MG, Gamble A, Williamson BN, Tamin A, Harcourt JL, Thornburg NJ, Gerber SI, Lloyd-Smith JO, de Wit E, Munster VJ (2020). Aerosol and Surface Stability of SARS-CoV-2 as Compared with SARS-CoV-1. N Engl J Med.

[CR37] Vuorinen V (2020). Modelling aerosol transport and virus exposure with numerical simulations in relation to SARS-CoV-2 transmission by inhalation indoors. Saf Sci.

[CR38] Wang Q, Huang R (2021). The impact of COVID-19 pandemic on sustainable development goals–A survey. Environ Res.

[CR39] World Health Organization (WHO) (2021, 25 May): Coronavirus disease (COVID-19) pandemic

[CR40] Yan J, Grantham M, Pantelic J, de Bueno Mesquita PJ, Albert B, Liu F, Ehrman S, Milton DK, Consortium E (2018). Infectious virus in exhaled breath of symptomatic seasonal influenza cases from a college community. Proc Natl Acad Sci U S A.

[CR41] Yan Y, Li X, Yang L, Yan P, Tu J (2020). Evaluation of cough-jet effects on the transport characteristics of respiratory-induced contaminants in airline passengers' local environments. Build Environ.

[CR42] Yao M, Zhang L, Ma J, Zhou L (2020). On airborne transmission and control of SARS-Cov-2. Sci Total Environ.

[CR43] Yip L, Finn M, Granados A, Prost K, McGeer A, Gubbay JB, Scott J, Mubareka S (2019). Influenza virus RNA recovered from droplets and droplet nuclei emitted by adults in an acute care setting. J Occup Environ Hyg.

[CR44] Zhao L, Qi Y, Luzzatto-Fegiz P, Cui Y, Zhu Y (2020). COVID-19: effects of environmental conditions on the propagation of respiratory droplets. Nano Lett.

